# Implementation of an Enhanced Recovery After Surgery (ERAS) Pathway Is Associated with Improved Short-Term Outcomes After Colorectal Surgery: A Retrospective Bi-Centre Study

**DOI:** 10.3390/jcm15155929

**Published:** 2026-07-29

**Authors:** Paolo Panaccio, Maira Farrukh, Maria Marino, Vincenzo Casolino, Giuseppe Di Martino, Pierluigi Di Sebastiano, Tommaso Grottola, Fabio Francesco di Mola

**Affiliations:** 1 General Surgery Unit, “F. Renzetti” Hospital, 66034 Lanciano, Italy; paolo.panaccio@gmail.com (P.P.); vincenzo.casolino@asl2abruzzo.it (V.C.); 2 Department of Innovative Technologies in Clinical Medicine and Dentistry, “G. d’Annunzio” University of Chieti-Pescara, 66100 Chieti, Italy; maira.farrukh@yahoo.it (M.F.); pierluigi.disebastiano@unich.it (P.D.S.); 3 Unit of Gastroenterology and Endoscopic Surgery, “G. Bernabeo” and “F. Renzetti” Hospital, 66034 Ortona-Lanciano, Italy; maria.marino12@ymail.com; 4 Department of Medicine and Ageing Sciences, “G. d’Annunzio” University of Chieti-Pescara, 66100 Chieti, Italy; giuseppe.dimartino@unich.it; 5 Unit of Epidemiology and Health Statistics, Local Health Authority of Pescara, 65124 Pescara, Italy; 6 Clinica Chirurgia II, Pierangeli Hospital, 65124 Pescara, Italy; tgrottoladoc@gmail.com

**Keywords:** Enhanced Recovery After Surgery (ERAS), colorectal surgery, minimally invasive surgery, laparoscopy, postoperative morbidity, anastomotic leakage, length of stay

## Abstract

**Background:** Enhanced Recovery After Surgery (ERAS) pathways have become the standard of care in elective colorectal surgery. However, implementation remains heterogeneous across institutions, and the relative contribution of ERAS pathways and minimally invasive surgery to improved postoperative outcomes remains uncertain. This study evaluated the association between ERAS implementation and short-term outcomes in two university-affiliated colorectal units with different levels of ERAS adoption. **Methods:** A retrospective bi-centre observational study was conducted, and comprised 802 consecutive patients who underwent elective colorectal resection between January 2016 and December 2024. Patients managed according to a standardized ERAS pathway (Group 1, *n* = 406) were compared with patients who received conventional perioperative care (Group 2, *n* = 396). Primary endpoints included postoperative morbidity, anastomotic leakage, and length of hospital stay (LOS). Secondary endpoints included mortality, readmission, postoperative complications, and hospitalization-related costs. Multivariable logistic regression was performed, adjusting for age, sex, ASA score, tumour stage, and tumour location. **Results:** Baseline demographic characteristics were largely comparable between groups, although patients in the conventional care group had a higher proportion of ASA III–IV status. Overall postoperative morbidity was significantly lower in the ERAS cohort (8.6% vs. 20.9%, *p* < 0.001), together with a lower incidence of anastomotic leakage (1.2% vs. 4.5%, *p* < 0.001). Median LOS was reduced from 9 to 8 days overall and, among patients who underwent laparoscopic surgery, from 6 to 4 days (*p* = 0.010). After multivariable adjustment, conventional perioperative management remained independently associated with higher postoperative morbidity (OR 2.62, 95% CI 1.60–4.09; *p* < 0.001) and anastomotic leakage (OR 1.92, 95% CI 1.01–4.98; *p* = 0.048). Mortality, surgical site infections, intra-abdominal abscesses, and other postoperative complications were comparable between groups. Based on regional reimbursement tariffs, ERAS implementation was associated with an estimated annual reduction of 567 hospital bed-days. **Conclusions:** Implementation of a standardized ERAS pathway was associated with reduced postoperative morbidity, lower anastomotic leakage rates, and shorter hospital stay after elective colorectal surgery. These benefits persisted after adjustment for major clinical confounders, supporting the effectiveness of standardized perioperative care. The greatest reduction in hospital stay was observed when ERAS was combined with minimally invasive surgery, emphasizing the complementary role of these strategies in optimizing postoperative recovery.

## 1. Introduction

Enhanced Recovery After Surgery (ERAS) is a multimodal, evidence-based perioperative care pathway designed to reduce the physiological stress response to surgery, maintain postoperative organ function, and accelerate patient recovery. The Enhanced Recovery After Surgery (ERAS) protocol was developed in 2005 in Edinburgh [1]. Since its introduction in colorectal surgery, ERAS protocols have progressively become the standard of care for elective colorectal procedures through the integration of preoperative optimization, standardized anesthetic management, minimally invasive surgery whenever feasible, multimodal analgesia, early oral nutrition, and early mobilization [1].

This pioneering study produced an integrated multimodal approach to perioperative care, resulting in an overall enhancement in recovery rates, a decrease in postoperative complications, reduced hospital stay, and early discharge after colorectal surgery.

The traditional factors responsible for delayed postoperative recovery, including inadequate pain control, prolonged postoperative ileus, immobility, and stress-induced organ dysfunction, represent the main targets addressed by ERAS pathways [2].

Although laparoscopic colorectal surgery has independently contributed to improved postoperative recovery by reducing surgical trauma and morbidity, its effects overlap with those of ERAS pathways, making the independent contribution of each component difficult to determine, particularly in observational studies [3,4].

The rate of postoperative recovery, especially in high-risk older patients, is determined by pain, stress-induced organ dysfunction, and limitations in conventional postoperative care. In 1995, Bardram attempted to provide “stress-free” colonic resection for neoplastic disease in eight elderly high-risk patients by a combination of laparoscopically assisted surgery, epidural analgesia, and early oral nutrition and mobilization [5].

A few years later, Kehlet et al. confirmed rapid recovery after open sigmoid resection using a multimodal rehabilitation programme, with emphasis on pain control with thoracic epidural anesthesia to control pain, resulting also in postoperative ileus and improved mobility [6].

Based on these principles, ERAS pathways have evolved into standardized multidisciplinary programmes aimed at reducing surgical stress, improving functional recovery, and decreasing postoperative complications after major colorectal surgery [7,8].

Enhanced recovery programmes or “fast-track” programmes have become an important focus of perioperative management after colorectal surgery [9].

Over the past two decades, substantial evidence has demonstrated that ERAS implementation is associated with shorter hospital stay, faster recovery, reduced postoperative morbidity, and improved resource utilization without increases in mortality or readmission rates [10,11,12].

Core items of the ERAS protocol include preoperative counselling, preoperative nutrition, avoidance of perioperative fasting and carbohydrate loading up to 2 h preoperatively, standardized anesthetic and analgesic regimens (epidural and non-opioid analgesia), and early mobilization, as shown in Table 1 [13]. These elements are integrated throughout the patient’s journey and provided by various hospital departments and specialists, including surgeons, nutritionists, nursing staff, and anesthesiologists.

Despite the increasing evidence supporting ERAS, implementation remains heterogeneous among institutions. Differences in organizational resources, multidisciplinary collaboration, staff training, and adherence to individual ERAS components represent important barriers to routine clinical application.

The ERAS literature shows substantial divergence concerning the composition of applied care bundles and the outcome parameters measured across various trials [14,15]. Furthermore, no universally accepted threshold of compliance has been established to define complete ERAS implementation, although higher adherence to ERAS components has been consistently associated with improved outcomes [13,16].

At present, no consensus has been reached on the number of items that must be applied for patients to be considered for fast-tracking, but robust evidence supports the application of ERAS protocols for the clinical management of colorectal surgical patients [13,16].

The aim of this study was to evaluate the association between ERAS implementation and short-term perioperative outcomes in patients undergoing elective colorectal cancer surgery treated in two different institutions affiliated with the University “G. D’Annunzio” of Chieti-Pescara.

We compared postoperative morbidity, anastomotic leakage, mortality, readmission rates, and length of hospital stay between patients managed according to an ERAS pathway and those receiving conventional perioperative care. In addition, we performed a simplified estimation of hospitalization-related costs. Given the retrospective observational design, this study was designed to evaluate associations between ERAS implementation and clinical outcomes rather than to establish causal effects.

## 2. Materials and Methods

### 2.1. Patient Selection

Building upon our prior experience with modified ERAS protocols at the Casa Sollievo della Sofferenza Hospital in San Giovanni Rotondo (FG, Italy), we applied a fast-track perioperative care programme in elective colorectal cancer surgery with the establishment of a dedicated database [17].

Since the introduction of the ERAS pathway in our surgical units in 2016, implementation progressively increased over time according to the availability of dedicated multidisciplinary resources and adherence to individual ERAS components. Patients were classified as ERAS (Group 1) when they were managed according to the institutional ERAS pathway and fulfilled the predefined mandatory perioperative items included in the protocol. Patients not managed according to the complete ERAS pathway were classified as conventional care patients (Group 2).

A retrospective review was performed on a prospectively collected bi-institutional database of patients who underwent either open or minimally invasive surgical treatment between January 2016 and December 2024 at the Clinica Chirurgica II, Pierangeli Hospital (Pescara, Italy) and the Unit of General Surgery, “F. Renzetti” Hospital (Lanciano, Italy).

Both facilities were affiliated with the “G. D’Annunzio” University of Chieti-Pescara (Italy). All surgeons involved in the study are highly skilled colorectal surgeons who have performed at least 50 minimally invasive colorectal resections within the last 5 years, and have worked at both centres. Surgical procedures and perioperative management followed standardized institutional protocols shared between the two centres (Table 1).

Data were collected using patient charts (medical records), anesthesia charts, operative reports, and pathology records. Follow-up was conducted via outpatient evaluation and telephone interview.

The primary endpoints of the study were postoperative morbidity, anastomotic leakage, and length of hospital stay. Secondary endpoints included postoperative mortality, readmission rate, reoperation rate, surgical site infection, intra-abdominal abscess, cardiopulmonary complications, and simplified estimation of hospitalization-related costs.

Inclusion criteria were laparoscopic or open colorectal surgery for benign or malignant pathology. For the ERAS group, patients were required to complete the predefined ERAS pathway according to institutional criteria (Table 1).

Exclusion criteria were ASA V patients and patients who underwent emergency colorectal surgery due to perforation, intestinal obstruction, or uncontrolled bleeding.

Collected variables included demographic characteristics (age, sex, and ASA score), tumour characteristics (location and pathological stage), operative approach (open or laparoscopic), type of colorectal resection performed, and postoperative outcomes. Although additional variables such as BMI, detailed comorbidities, neoadjuvant treatment, and conversion from laparoscopy to open surgery were available for a proportion of patients, they were not consistently recorded across both institutional databases throughout the study period and were therefore not included in the comparative analyses.

The collected postoperative outcomes included 30-day readmission rates, reoperation rates, postoperative morbidity, and mortality.

### 2.2. Operative Technique

Regarding minimally invasive approaches, a traditional medial-to-lateral approach was routinely followed in both right-sided and left-sided resections. The splenic flexure was mobilized for all rectal resections and, when necessary, for sigmoid colectomies. Conversion from laparoscopic to open surgery was recorded and considered a perioperative outcome. A conversion to open surgery was performed in cases of intraoperative complications such as bleeding or when dissection could not be safely completed laparoscopically. The specimen was extracted by a Pfannenstiel incision through a wound protection device or an endobag. Anastomoses to the rectum were performed with circular staplers (Knight–Griffen anastomosis), whereas ileocolic anastomoses were mechanical and intracorporeal. Defunctioning ileostomies were performed in cases of ultra-low rectal resection or high-risk anastomoses. Transanal total mesorectal excision (TaTME) resections were carried out in selected patients with mid–low rectal cancer.

### 2.3. Enhanced Recovery Programme Protocol

Following previously established evidence, a multidisciplinary institutional committee comprising surgeons, anesthesiologists, nursing staff, and dieticians developed a standardized clinical pathway. The ERAS protocol included 23 perioperative care items according to current ERAS recommendations summarized in Table 1. Compliance was evaluated through prospective recording of completed ERAS items within the institutional database.

### 2.4. Group 1 (ERAS Group)

Nutritional counselling was provided and moderate aerobic physical activity was recommended according to current evidence [18,19]. Energy intake, proteins, and lipids remained unchanged until admission. Only fibre consumption was reduced three days before admission according to low-fibre diet recommendations [20]. After admission, patients were managed according to ERAS principles, including no solid food after midnight, carbohydrate loading with maltodextrins, and clear fluids up to 2 h before surgery. Antibiotic prophylaxis consisted of a single dose of ampicillin/sulbactam (3 g) and metronidazole (0.5 g) 30 min before surgery, with re-administration when procedures exceeded 3 h. Thromboembolic prophylaxis consisted of low-molecular-weight heparin and compression stockings until discharge. Extended prophylaxis for 2 or 4 weeks was administered in patients undergoing colorectal cancer surgery. Mechanical bowel preparation was reserved exclusively for rectal cancer patients. Intraoperative fluid administration was restricted to 1500 mL except in cases of major blood loss. Postoperatively, patients were transferred either to the Intensive Care Unit according to ASA status and anesthesiologist evaluation or to the surgical ward. The nasogastric tube was removed at the end of surgery, and oral nutrition was progressively resumed starting from the evening of surgery. Early mobilization was encouraged from postoperative day 1.

### 2.5. Group 2 (Non-ERAS)

Patients in the conventional care group received standard perioperative management, including bowel preparation according to local practice, unrestricted intraoperative and postoperative fluid administration, and conventional analgesic strategies. Patients in Group 2 were treated before complete ERAS implementation or when the complete ERAS pathway was not applied. The nasogastric tube was removed after clinical recovery of bowel function. Discharge criteria remained comparable between groups. Since January 2025, the ERAS pathway has been applied routinely to all colorectal resections. Patients treated after this date were not included in the comparative analysis because the objective of the present study was to compare historical ERAS and conventional care cohorts during the implementation phase.

### 2.6. Definition of Postoperative Outcomes

Discharge was considered feasible from postoperative day 5 provided that clinical criteria were fulfilled. Discharge criteria included:

- Adequate pain control with oral therapy;
- Absence of nausea or vomiting;
- Tolerance of solid food;
- Regular passage of flatus and/or stool;
- Absence of infection signs;
- Adequate mobilization;
- Patient agreement.

For patients requiring a stoma, standardized education was provided before discharge. Anastomotic leakage was identified based on clinical findings, radiological evidence, and/or intraoperative confirmation. An intra-abdominal abscess was defined as an infected fluid collection within the abdominal or pelvic cavity. Surgical site infection was defined according to clinical criteria requiring antibiotic therapy and/or wound treatment. Hemorrhage was defined as postoperative bleeding requiring transfusion and/or surgical intervention. Cardiorespiratory complications included clinically relevant atrial fibrillation, heart failure, pneumonia, or respiratory complications requiring pharmacological treatment.

### 2.7. Statistical Analysis

Statistical analysis was performed using SPSS (version 13). Continuous variables were expressed as the median and range and compared using the Mann–Whitney U test. Categorical variables were reported as frequency and percentages and compared using the Chi-square or Fisher’s exact test. To evaluate the association between complications and ERAS, multivariable logistic regression models were performed. All models were adjusted for age, gender, ASA score, tumour side, and tumour stage. Associations were expressed as odds ratios (ORs) with relative 95% confidence intervals (95% CIs). A *p*-value of less than 0.05 was considered statistically significant.

## 3. Results

A total of 802 patients undergoing elective colorectal surgery for cancer were collected. Perioperative data from 406 consecutive patients undergoing colorectal resection via the fast-track protocol (Group 1) were analyzed and compared with 396 non-ERAS resections (Group 2) (Table 2).

Both groups were further stratified by surgical approach: in Group 1, 189 patients underwent open resection and 217 underwent laparoscopic surgery. In Group 2, 225 patients underwent open surgery and 171 underwent laparoscopic procedures. This difference in surgical approach between the two groups was statistically significant (*p* = 0.004) (Table 3).

With regard to the comorbidities of patients enrolled in the study, no significant differences were reported for preoperative risk in ASA groups I–II (according to the ASA score). Baseline demographic characteristics were generally comparable between groups, except for the ASA score. Variables such as BMI, detailed comorbidities, neoadjuvant treatment, and laparoscopic conversion were not consistently available in both institutional databases and were therefore not included in the comparative analysis (Table 2 and Table 3). The median age was 69 years (range: 33–93) in Group 1 and 68 years (range: 36–94) in Group 2.

ASA III or IV patients were more often assigned to the non-ERAS group, as they exhibited either more frequent postoperative complications and/or reduced compliance with the full set of protocol requirements. Overall, 75% of patients demonstrated full adherence to all ERAS protocol requirements.

No significant differences were found between the two groups regarding surgical technique (open vs. laparoscopic) or tumour location:

- Tumour location: colon (57%), rectum (27%), rectosigmoid (16%), bifocal (nine cases), and local or anastomotic recurrence (one case) in ERAS (Group 1).
- Tumour location: colon (54.2%), rectum (30.3%), rectosigmoid (12.9%), bifocal (six cases), and local or anastomotic recurrence (one case) in non-ERAS (Group 2).

The median length of stay was 8 days (range: 5–125) for Group 1 and 9 days (range: 5–74) for Group 2 (*p* < 0.05) (Table 4).

Regarding postoperative outcomes, a significant difference was observed in the overall complication rates between the two cohorts (Table 5 and Table 6). Patients in Group 1 experienced a substantially lower incidence of general complications compared to Group 2 [35/406 (8.6%) vs. 83/396 (20.9%); *p* < 0.001] (Table 7).

Specifically, the analysis of major surgical complications revealed that anastomotic leakage occurred in five patients (1.2%) in Group 1, whereas 18 patients (4.5%) were affected in Group 2. This three-fold reduction in the ERAS group was statistically significant (*p* < 0.001). No other major surgical site-related discrepancies reached the same level of significance. Although the 30-day readmission rate was lower in Group 1 (1% vs. 2.7%), this difference did not reach statistical significance (*p* = 0.071) (Table 7). Multivariable logistic regression analysis was performed to adjust for potential confounding factors, including age, sex, ASA score, tumour stage, and tumour location (Table 8). After adjustment, patients in Group 2 had more than twice the odds of developing overall postoperative morbidity compared with those in Group 1 (OR 2.62, 95% CI 1.60–4.09; *p* < 0.001). Likewise, the odds of anastomotic leakage remained significantly higher in Group 2 (OR 1.92, 95% CI 1.01–4.98; *p* = 0.048). No statistically significant differences were observed for postoperative mortality, intra-abdominal abscess, hemorrhagic complications, pulmonary complications, surgical site infection, or other postoperative events.

The specific complications for each group are listed below:

- Group 1 Specific Complications: Anastomotic leak 2.2%, hemorrhage 2.2%, abscess 2.8%, surgical site infection (SSI) 3.2%, respiratory distress 2.2%, pulmonary complications 0.5%, and others 2.7%.
- Group 2 Specific Complications: Anastomotic leak 4.5%, hemorrhage 3.5%, abscess 4.5%, heart failure 0.7%, intestinal ischemia 0.7%, SSI 4%, respiratory distress 1.7%, pulmonary complications 1.7%. Mortality was 1.4% in Group 1 vs. 3.3% in Group 2 (Table 5 and Table 6).

Mean time of follow-up was 14.6 ± 6.6 months for the non-ERAS and 6.6 ± 3.4 months for the ERAS group (*p* < 0.0001). The significantly shorter follow-up in the ERAS group (6.6 vs. 14.6 months) primarily reflects its recent full implementation.

In the laparoscopic subgroup, postoperative morbidity was generally low in both cohorts (Table 8). Anastomotic leakage occurred in three patients (1.3%) in Group 1 and seven patients (4.1%) in Group 2; although the incidence was numerically lower in Group 1, the difference did not reach statistical significance (*p* = 0.110). Similarly, no significant differences were observed in the rates of intra-abdominal abscesses (1.8% vs. 1.3%, *p* = 0.224), surgical site infections (1.0% vs. 1.7%, *p* = 0.358), or other postoperative complications (2.8% vs. 5.2%, *p* = 0.240). In contrast, postoperative hemorrhagic complications, including intra-abdominal and gastrointestinal bleeding, were significantly less frequent in Group 1 than in Group 2 (1.3% vs. 5.26%, *p* = 0.035). Mortality was extremely low, with no deaths reported in Group 1 and one postoperative death in Group 2 (Table 9).

Among the 229 patients undergoing rectal resection, Group 1 was characterized by a markedly higher adoption of laparoscopic surgery than Group 2 (78.0% vs. 21.7%, *p* < 0.001), whereas the proportion of restorative procedures was comparable between cohorts (78.0% vs. 77.5%, *p* = 0.930). Patients treated in Group 1 experienced a lower incidence of overall postoperative complications (23.9% vs. 31.7%) and postoperative mortality (0.9% vs. 5.0%), although these differences did not reach statistical significance. Likewise, no significant differences were observed in the rates of intra-abdominal abscess, anastomotic leakage, postoperative hemorrhage, surgical site infection, bowel ischemia, or reoperation. Pulmonary complications and postoperative respiratory distress were numerically less frequent in Group 1 than in Group 2 (0.9% vs. 4.2% and 0.9% vs. 5.0%, respectively), suggesting a trend toward improved postoperative recovery in the cohort managed predominantly with minimally invasive surgery and ERAS principles (Table 10).

### Cost-Effectiveness Analysis

Based on the official tariffs for hospital care in the Abruzzo Region (as detailed in Regional Council Resolution—DGR n. 929 and n. 930 of 20 December 2023), the estimated daily ward cost for surgical patients ranges between €600 and €800. Consequently, implementing the ERAS protocol across the entire control cohort (*n* = 396) would have yielded a projected saving of 567 bed-days per year, equating to a total expenditure decrease of €340,200 to €453,600.

## 4. Discussion

The present study evaluated the association between the implementation of an Enhanced Recovery After Surgery (ERAS) pathway and short-term outcomes in 802 patients who underwent elective colorectal cancer surgery. Our findings suggest that patients managed according to an ERAS pathway experienced a shorter hospital stay and lower postoperative morbidity compared with patients who received conventional perioperative care. However, given the retrospective observational design of the study, these results should be interpreted as associations rather than evidence of a direct causal effect of ERAS implementation.

One of the main objectives of ERAS pathways is to accelerate functional recovery and reduce unnecessary delays in postoperative care. Our data show a statistically significant reduction in the median length of stay, decreasing from 9 days in Group 2 to 8 days in Group 1. Although the absolute difference was limited, even small reductions in length of stay may represent relevant improvements in resource utilization when applied to large surgical populations. This was achieved with a protocol compliance rate exceeding 75%, supporting the feasibility of implementing standardized perioperative pathways in routine clinical practice. The reduction in postoperative morbidity represents another important finding of our study. Group 1 experienced a lower rate of overall complications compared with Group 2 (8.6% vs. 20.9%; *p* < 0.001). Several mechanisms may contribute to this observation, including optimized perioperative fluid management, standardized analgesic strategies, early mobilization, early nutritional support, and multidisciplinary postoperative care [18,19,20,21]. However, it should be emphasized that the reduction in complications cannot be attributed exclusively to ERAS, as other relevant differences between groups may have influenced these outcomes. Anastomotic leakage is a multifactorial complication influenced by several patient- and procedure-related factors, including tumour location, nutritional status, comorbidities, ASA score, surgical technique, surgeon experience, and perioperative management [22,23]. The incidence of anastomotic leakage was lower in the ERAS group (1.2% vs. 4.5%; *p* < 0.001). Although ERAS components such as optimized fluid management, avoidance of unnecessary drains, early recognition of complications, and standardized postoperative care may contribute to improved recovery, the observed difference should be interpreted cautiously because the ERAS cohort also differed in surgical approach and baseline characteristics.

To account for potential baseline differences between the two cohorts, a multivariable logistic regression analysis was performed adjusting for age, sex, ASA score, tumour stage, and tumour location. After adjustment, patients treated in Group 2 remained at a significantly higher risk of overall postoperative morbidity (OR 2.62, 95% CI 1.60–4.09; *p* < 0.001), suggesting that the observed reduction in complications in Group 1 was not solely explained by differences in patient characteristics or disease severity. These findings support the hypothesis that standardized perioperative management, together with greater implementation of minimally invasive surgery, may have contributed to improved short-term postoperative outcomes.

Interestingly, anastomotic leakage remained independently associated with Group 2 (OR 1.92, 95% CI 1.01–4.98; *p* = 0.048). Although the absolute number of leakage events was low, this observation is clinically relevant because anastomotic failure represents one of the most severe complications following colorectal surgery. Nevertheless, given the relatively limited number of events, this result should be interpreted with caution and considered hypothesis-generating rather than definitive.

No independent association was observed for postoperative mortality, intra-abdominal abscesses, surgical site infection, postoperative hemorrhage, pulmonary complications, or other postoperative event after multivariable adjustment. This likely reflects the overall low incidence of these complications and the limited statistical power for individual endpoints rather than the absence of clinically meaningful differences.

Overall, the multivariable analysis reinforces the robustness of the primary findings by demonstrating that the reduction in overall morbidity persisted after adjustment for relevant clinical confounders. These results further support the concept that the combination of ERAS implementation and a standardized minimally invasive surgical pathway may contribute to improved postoperative recovery in patients undergoing colorectal cancer surgery.

Another relevant finding of this study was the higher proportion of laparoscopic procedures performed in the ERAS group, particularly among patients undergoing rectal surgery (78% vs. 22%). Minimally invasive surgery is independently associated with reduced surgical trauma, lower inflammatory response, faster gastrointestinal recovery, and reduced postoperative morbidity [4]. Therefore, the improved outcomes observed in the ERAS cohort may reflect the combined effect of ERAS implementation and increased adoption of minimally invasive techniques rather than the isolated effect of ERAS alone.

Importantly, the markedly different adoption of laparoscopic surgery between the two participating centres, particularly among patients undergoing rectal resection (78% vs. 22%), represents one of the major limitations of the present study. Because ERAS implementation and minimally invasive surgery were closely associated within our cohort, it is not possible to completely disentangle the independent contribution of each intervention to the observed clinical outcomes. Although multivariable adjustment was performed for several relevant clinical confounders, residual confounding related to surgical approach cannot be excluded. Consequently, the observed benefits should be interpreted as the combined effect of standardized ERAS implementation and greater adoption of minimally invasive surgery rather than the isolated effect of ERAS alone.

Future studies with adequate adjustment for surgical approach are required to clarify the independent contribution of each factor. The lower incidence of pulmonary complications and surgical site infections observed in the ERAS group may also be partially explained by this combination of factors. Reduced incision size, earlier mobilization, opioid-sparing analgesia, and optimized perioperative care are all elements that may contribute to improved postoperative recovery. Nevertheless, the retrospective design of our study does not allow determination of the relative contribution of each individual intervention. The mortality rate was lower in the ERAS group (1.4% vs. 3.3%), although this difference did not reach statistical significance. This finding suggests that ERAS implementation did not negatively affect patient safety; however, the study was not powered to detect differences in rare outcomes such as postoperative mortality.

The present study highlights several important limitations related to patient selection and study design. First, this was a retrospective observational study performed in two different institutions, and the ERAS and conventional care groups were not fully comparable. Patients in the conventional care group had a higher proportion of ASA III–IV status, suggesting a greater burden of comorbidity and potentially higher baseline surgical risk. In addition, differences in hospital organization, surgical expertise, and local implementation strategies may have contributed to the observed outcomes. Second, the study period included several years during which surgical techniques, perioperative management, anesthetic protocols, and institutional experience progressively evolved. Therefore, temporal bias cannot be excluded. The shorter follow-up duration observed in the ERAS group reflects the more recent implementation of the pathway and may have influenced the detection of late complications or readmissions.

Furthermore, several potentially relevant variables, including BMI, detailed comorbidity assessment, neoadjuvant treatment, and laparoscopic conversion rate, were not consistently available throughout the entire study period in both institutional databases. Consequently, these variables could not be included in the baseline comparison or multivariable models and may have contributed to residual confounding.

Previous studies have suggested that ERAS pathways may reduce healthcare expenditure mainly through shorter hospitalization and reduced postoperative complications [24,25,26]. However, the present analysis should be considered a simplified estimation based primarily on hospital length of stay rather than a formal cost-effectiveness analysis. The costs associated with ERAS implementation, including multidisciplinary coordination, staff training, nutritional assessment, physiotherapy, patient education, and organizational resources, were not included. Overall, our findings support the feasibility of implementing an ERAS pathway in elective colorectal surgery and suggest an association with improved short-term postoperative outcomes. Nevertheless, because ERAS implementation occurred together with other changes in clinical practice, particularly the increased use of minimally invasive surgery and differences in patient characteristics, the observed benefits should be interpreted cautiously. Prospective multicenter studies with standardized ERAS compliance assessment and appropriate adjustment for confounding factors are needed to define the independent effect of ERAS on colorectal surgical outcomes.

## Figures and Tables

**Table 1:** Care plan for patients in the fast-track (ERAS) surgery programme.

Timing	Intervention
Preoperative Day 1	Normal oral nutrition until midnight. No anesthetic premedication.
Day of Surgery	Analgesia via elastomeric pump (ketoprofen 960 mg, tramadol 600 mg, ranitidine 450 mg, metoclopramide 90 mg in saline). No nasogastric tube; if used, removed immediately after extubation. Warm intravenous fluids, upper and lower body warming devices. Goal-directed fluid therapy (CVP < 5 mmHg, max 50 mL/h). Transversus abdominis plane (TAP) block. Single-shot antibiotic and anti-thrombotic prophylaxis.
Postoperative Day 1	Return to surgical ward. Mobilization (at least four times per day). Resumption of oral intake with liquids and solids as tolerated. Continued analgesia with elastomeric pump. Paracetamol 1000 mg every 6 h. Intravenous metoclopramide.
Postoperative Day 2	Discontinuation of elastomeric pump and initiation of oral NSAIDs. Mobilization (at least four times per day). Oral metoclopramide. Light or full diet as tolerated. Discharge if feasible.
Postoperative Day 3	Unrestricted mobilization.
Postoperative Day 4	Normal diet and discharge.

**Table 2:** Demographic and perioperative data.

Variabile	Group 1 N = 406	Group 2 N = 396	
Age, y (median, range)	69 (33–93)	68 (36–94)	0.849
Sex			0.663
Male (M)	242 (59.7%)	242 (61.1%)	
Female (F)	164 (40.3%)	154 (38.9%)	
ASA Score			<0.001
I	139 (34%)	79	
II	217 (54%)	141 (35.6%)	
III	50 (12%)	161	
IV	0	15	
Tumour side			0.011
Colon	230 (57%)	215 (54.2%)	
Sigmoid colon	67 (16%)	51 (12.9%)	
Rectum (overall)	109 (27%)	120 (30.3%)	
High	20	22	
Medium	25	51	
Low	10	47	
Multifocal	9	10	
Recurrence	1	0	
Tumour Stage			0.899
Dukes A	67	65	
Dukes B	202	203	
Dukes C	137	128	
Dukes D	0	0	

**Table 3:** Surgical procedure.

	Group 1	Group 2
Open surgery	189	225
Proctectomy with J pouch	2	0
Subtotal colectomy	2	6
Total colectomy	2	2
Right hemicolectomy	83	96
Left hemicolectomy	19	33
Sigmoidectomy	10	6
Upper anterior resection	20	19
Lower anterior resection	19	32
Ultra-low rectal resection (intersphincteric)	5	0
Hartmann resection	7	6
Miles (abdominoperineal resection)	9	12
Other resection (e.g., segmental)	11	13
Laparoscopic surgery	217	171
Left hemicolectomy	20	42
Splenic flexure resection	42	7
Right hemicolectomy	50	60
Sigmoidectomy	50	36
Upper anterior resection	20	19
Lower anterior resection	28	0
Miles/Ta-TME	5	6
Ultra-low rectal resection	2	1
Sample size	406	396

Ta-TME: Transanal total mesorectal exision.

**Table 4:** Length of stay (LOS).

Open Surgery	Group 1 (*n* = 406)	Group 2 (*n* = 396)
Discharge (days)	8 (range 5–125)	9 (range 5–74)
Readmission (n)	4 (1%)	11 (2.7%)
Laparoscopic surgery		
Discharge (days)	4 gg (2–28)	6 gg (5–21)
Readmission (n)	2 (1%)	4 (2.3%)

**Table 5:** Short-term outcomes in overall patient population.

	Group 1 (*n* = 406)	Group 2 (*n* = 396)
Morbidity	35 (8.6%)	83 (21.1%)
Mortality	5 (1.4%)	13 (3.3%)
Intrabdominal abscess	11 (2.7%)	18 (4.5%)
Anastomotic leak	9 (2.2%)	18 (4.5%)
Heart failure	0	3 (0.7%)
Hemorrhage (intrabdominal or gastroenteric)	9 (2.2%)	14 (3.5%)
Liver failure	0	0
Bowel ischemia	0	3 (0.7%)
Pulmonary complications	2 (0.5%)	7 (1.7%)
Surgical site infection (SSI)	13 (3.2%)	16 (4%)
Other	11 (2.7%)	7 (1.7%)

**Table 6:** Short-term outcomes in laparoscopic surgery.

	Group 1 (*n* = 217)	Group 2 (*n* = 171)
Anastomotic leak	3 (1.3%)	7 (4.1%)
Hemorrhage (intrabdominal or gastroenteric)	3 (1.3%)	9 (2.2%)
Intrabdominal abscess	4 (1.8%)	5 (1.3%)
SSI	2 (1%)	3 (1.7%)
Other	6 (2.8%)	9 (5.2%)
Mortality	0	1 (0.05%)

**Table 7:** Statistical analysis.

	Group 1 (*n* = 406)	Group 2 (*n* = 396)	*p*-Value
Age	69 (33–93)	68 (36–94)	0.478
Sex (M/F)	242/164	242/154	0.189
ASA score			<0.001
I	139 (34%)	79 (19.9%)	
II	217 (54%)	141 (35.6%)	
III	50 (12%)	161 (40.6%)	
IV	0	15 (3.8%)	
Complications	35 (8.6%)	83 (20.9%)	<0.001
Anastomotic leak	5 (1.2%)	18 (4.5%)	<0.001
Readmission	6 (1.5%)	11 (2.8%)	0.201
Length of Stay (LOS) (days), open surgery	8 (5–125)	9 (5–74)	0.115
LOS (days), laparoscopic	4 d (2–28)	6 d (5–21)	0.010

**Table 8:** Multivariable logistic regression models for associations between groups and short-term outcomes.

	OR (IC95%)	*p*-Value
Morbidity	2.62 (1.60–4.09)	<0.001
Mortality	2.18 (0.72–6.51)	0.165
Intrabdominal abscess	1.80 (0.68–4.28)	0.162
Anastomotic leak	1.92 (1.01–4.98)	0.048
Heart failure	Not applicable	
Hemorrhage (intrabdominal or gastroenteric)	0.99 (0.61–4.06)	0.779
Liver failure	Not applicable	
Bowel ischemia	Not applicable	
Pulmonary complications	3.18 (0.61–16.03)	0.170
SSI	1.15 (0.51–2.62)	0.723
Other	0.47 (0.16–1.34)	0.159

All models were adjusted for age, sex, ASA score, tumour stage, and tumour side. The ERAS group (Group 1) was considered as the reference in the multivariable logistic model.

**Table 9:** Complications in the laparoscopic subgroup.

	Group 1 (*n* = 217)	Group 2 (*n* = 171)	*p*-Value
Anastomotic leak	3 (1.3%)	7 (4.1%)	0.110
Hemorrhage (intrabdominal or gastroenteric)	3 (1.3%)	9 (5.2%)	0.035
Intrabdominal abscess	4 (1.8%)	5 (2.9%)	0.224
SSI	2 (1%)	3 (1.7%)	0.358
Other	6 (2.8%)	9 (5.2%)	0.240
Mortality	0	1 (0.5%)	na

**Table 10:** Demographic and technical information and postoperative outcomes in rectal resections.

	Group 1 (*n* = 109)	Group 2 (*n* = 120)	*p*-Value
Surgical approach			
Open surgery	24 (22.0%)	94 (78.3%)	<0.001
Laparoscopic surgery	85 (78.0%)	26 (21.7%)	<0.001
Patient characteristics			
Male sex	75 (68.8%)	76 (63.3%)	0.38
Female sex	34 (31.2%)	44 (36.7%)	
Operative data			
Restorative anastomosis	85 (78.0%)	93 (77.5%)	0.930
Postoperative outcomes			
Overall complications	26 (23.9%)	38 (31.7%)	0.19
Mortality	1 (0.9%)	6 (5.0%)	0.12 *
Morbidity	22 (20.2%)	28 (23.3%)	0.57
Intra-abdominal abscess	9 (8.3%)	10 (8.3%)	1.000
Total anastomosis	85 (77%)	93 (77.5%)	0.930
Anastomotic leak	2 (1.8%)	4 (3.3%)	0.48 *
Heart failure	0	1 (0.8%)	1.000 *
Postoperative hemorrhage	1 (0.9%)	3 (2.5%)	0.62 *
Liver failure	0	0	NA
Bowel ischemia	2 (1.8%)	1 (0.8%)	0.61 *
Pulmonary complications	1 (0.9%)	5 (4.2%)	0.22 *
Respiratory distress	1 (0.9%)	6 (5.0%)	0.12 *
Surgical site infection	1 (0.9%)	4 (3.3%)	0.37 *
Other complications	2 (1.8%)	0	0.23 *
Reoperation	1 (0.9%)	2 (1.7%)	1.000 *

Values are expressed as n (%). * Fisher’s exact test.

## Data Availability

The original contributions presented in the study are included in the article.
